# Virtual Reality Cognitive Training Among Individuals With Alcohol Use Disorder Undergoing Residential Treatment: Pilot Randomized Controlled Trial

**DOI:** 10.2196/18482

**Published:** 2021-01-29

**Authors:** Pedro Gamito, Jorge Oliveira, Marcelo Matias, Elsa Cunha, Rodrigo Brito, Paulo Ferreira Lopes, Alberto Deus

**Affiliations:** 1 School of Psychology and Life Sciences Lusófona University Lisboa Portugal; 2 Digital Human-Environment Interaction Lab Lusófona University Lisboa Portugal; 3 Casa de Saúde do Telhal Instituto São João de Deus Sintra Portugal

**Keywords:** alcohol use disorder, cognitive training, virtual reality

## Abstract

**Background:**

Alcohol use disorder (AUD) has been associated with diverse physical and mental morbidities. Among the main consequences of chronic and excessive alcohol use are cognitive and executive deficits. Some of these deficits may be reversed in specific cognitive and executive domains with behavioral approaches consisting of cognitive training. The advent of computer-based interventions may leverage these improvements, but randomized controlled trials (RCTs) of digital interactive-based interventions are still scarce.

**Objective:**

The aim of this study is to explore whether a cognitive training approach using VR exercises based on activities of daily living is feasible for improving the cognitive function of patients with AUD undergoing residential treatment, as well as to estimate the effect size for this intervention to power future definitive RCTs.

**Methods:**

This study consisted of a two-arm pilot RCT with a sample of 36 individuals recovering from AUD in a therapeutic community; experimental group participants received a therapist-guided, VR-based cognitive training intervention combined with treatment as usual, and control group participants received treatment as usual without cognitive training. A comprehensive neuropsychological battery of tests was used both at pre- and postassessments, including measurement of global cognition, executive functions, attention, visual memory, and cognitive flexibility.

**Results:**

In order to control for potential effects of global cognition and executive functions at baseline, these domains were controlled for in the statistical analysis for each individual outcome. Results indicate intervention effects on attention in two out of five outcomes and on cognitive flexibility in two out of six outcomes, with effect sizes in significant comparisons being larger for attention than for cognitive flexibility. Patient retention in cognitive training was high, in line with previous studies.

**Conclusions:**

Overall, the data suggest that VR-based cognitive training results in specific contributions to improving attention ability and cognitive flexibility of patients recovering from AUD.

**Trial Registration:**

ClinicalTrials.gov NCT04505345; https://clinicaltrials.gov/show/NCT04505345

## Introduction

Alcohol is a psychoactive substance that acts on the central nervous system, leading to dependence while causing severe physical, mental, and social problems [1]. Around 400 million people worldwide have problems related to excessive alcohol use, which is the third cause of death globally [2]. Alcohol abuse is also associated with diverse brain modifications [3], both at the structural and functional levels related to long-term, transient, or compensatory effects of alcohol [4], which have an impact on the integrity of the prefrontal brain cortex [5], causing deficits across a wide range of cognitive skills. These effects are particularly evident at the level of executive dysfunction, including attention, inhibitory control, behavioral control [3], verbal fluency, and decision making, similar to the effects of other substances, such as cannabis and cocaine [6]. Alcohol abuse in extreme cases may also lead to Korsakoff syndrome, which is characterized as an irreversible condition of anterograde amnesia [7].

These impairments at the brain level may disrupt behavior in such a way that individuals become overreactive to external cues related to the substance; this causes them to be unable to control substance-seeking behaviors and to make long-term decisions [8], which also affects treatment outcomes [9].

However, cognitive deficits resulting from chronic alcohol consumption are usually at least partially reversed during inpatient recovery periods, mostly as a result of abstinence [10]. While alcohol abuse is associated with morphological changes and reduced volume in multiple brain regions, mostly in the frontal lobe [5], abstinence from alcohol by patients recovering from alcohol use disorder (AUD) has been associated with significant recoveries in brain volume. The brain volume in these patients increases with the duration of the abstinence period; they also experience cognitive recoveries in speed and processing and shorter reaction times to stimuli [11]. Well-established treatments for alcohol dependence are, therefore, mostly designed with a focus on patients maintaining alcohol abstinence. Nevertheless, there is also agreement that the positive effects of abstinence at the cognitive level can be enhanced by cognitive interventions specifically designed to recover functions most compromised by alcohol abuse [12]; tests of the effectiveness of these interventions in the context of substance use disorders (SUDs) have been growing in recent years [13]. These cognitive interventions may be delivered in different formats. Cognitive stimulation typically involves group interventions for improvements not only in cognition but also socialization and does not focus on particular cognitive abilities. In contrast, cognitive training is a systematic training approach in which cognitive tasks are more focused on specific cognitive functions [14]. Its aim is to recover individuals’ potential and minimize the impact of brain lesions via a set of programmed behavioral activities involving different cognitive functions [15]. For instance, Yohman and colleagues [16] found that patients who underwent classic cognitive training that focused on memory and problem-solving abilities showed greater improvements in the cognitive areas related to those abilities, but not to memory, than did control patients. Goldstein and colleagues [17] found that AUD participants exhibited significantly increased levels of perceptual and visuospatial skills, speed of information processing, and attention.

Meanwhile, recent technological progress has allowed new solutions for cognitive training based on the use of computerized systems to be developed. This trend is known as computerized cognitive training (CCT) and includes a growing number of systems available for cognitive training in different clinical contexts. An early study involving CCT did not show significant cognitive improvements from this treatment among individuals in residential treatment for AUD in comparison to controls [18], but more recent studies suggest that there are specific contributions from CCT for improving cognition in AUD patients. For instance, Fals-Stewart and Lam [12] found that individuals recovering from SUD, including abuse of alcohol, who underwent cognitive intervention within residential treatment programs reported better treatment outcomes than controls who underwent computer typing training and treatment as usual. Individuals in the experimental group reported being more involved both in the treatment and in the recovery program and stayed abstinent for longer periods of time; they also reported improved social and family behaviors. Two more recent studies using CCT have found specific cognitive improvements among patients in recovery programs for alcohol or other substances, with effects in executive functions [19], attention, delayed memory, and working memory [20].

A recent systematic review of cognitive training in AUD recovery suggests that these approaches may be useful to promote cognitive functioning on top of improvements due to mere abstinence. However, the available data with AUD individuals only provide evidence for near-transfer effects to very similar tasks, with no evidence regarding far-transfer effects to dissimilar tasks or to everyday functionality [13].

Some authors argue that virtual reality (VR)–based cognitive training is an especially ecologically valid form of CCT because it includes exercises that mirror everyday life activities and those that involve similar demands to those of everyday living [21]. For example, using VR-based serious games makes it possible to replicate different activities of daily living, such as tasks related to hygiene, having breakfast, choosing clothes, and going shopping, thus providing a function-led cognitive training approach [22], which may promote far-transfer effects of training to everyday living activities [23]. VR-based serious games have other advantages over traditional methods: immediate dynamic feedback, repeated practice, no physical consequences after an error, the setting, the fact that the task can be customized to the patient, and the fact that training involves progressive learning [19].

Thus, previous research suggests that there are positive effects of computer-based cognitive training, but the specific contributions of VR-based cognitive training reproducing everyday life activities have not yet been demonstrated. In particular, randomized controlled trials (RCTs) have been lacking. This study builds on previous research but proposes a pilot RCT to estimate the effects of a VR-based cognitive intervention on patients with AUD at the level of memory, attention functions, and executive functions; this approach will help in reducing biases associated with previous noncontrolled studies [13], while contributing to the estimation of effect sizes to determine necessary power for future definitive RCTs.

## Methods

### Trial Design

The study design consisted of a two-arm pre-post RCT in which participants were assigned either to an experimental group that underwent VR-based cognitive training combined with treatment as usual in residential community rehabilitation or to a passive control group comprising treatment as usual but without VR-based cognitive training. This trial consisted of an open-label RCT in which patients, researchers, assessors, and therapists were not blinded to group allocation. The allocation ratio was 1:1. Both groups underwent the same treatment program for alcohol recovery that is administered to all inpatients in residential treatment. Random allocation was concealed prior to the start of the study and was based on simple randomization after baseline assessment with random number generation in Microsoft Excel, Office 365. The intervention model consisted of a parallel design. The trial was retrospectively registered at ClinicalTrials.gov (NCT04505345).

### Recruitment

The sample was recruited from September 2017 to May 2018 at a clinic for recovery from AUD, *Casa de Saúde do Telhal*, in the Lisbon region of Portugal. Recruitment was conducted during the psychological appointment of each patient’s first week of treatment. Treatment for recovery from AUD in this institution is based on a 4-week residential treatment program following the Minnesota Model. To be included in the study, participants had to be at least 18 years old, with normal or corrected-to-normal vision and hearing, without a history of psychiatric or neurological disorders, and attending the inpatient program at the institution where the data were collected. Exclusion criteria were the presence of a psychosis episode during the program and withdrawal from the inpatient program. These criteria were checked by the clinician who conducted patient recruitment.

### Outcomes

The outcomes of this study were selected from well-established neuropsychological tests. Primary outcomes were based on general cognitive functioning and executive functions, while secondary outcomes were based on specific cognitive tests for memory, attention, and cognitive flexibility.

#### Global Cognition

The Montreal Cognitive Assessment (MoCA) [24] was developed as a rapid tool for cognitive screening and has been validated for the Portuguese population [25]. The average duration of assessment completion ranges from 10 to 15 minutes, with no specified time limit. It consists of 11 items assessing cognitive domains, such as visuospatial orientation, naming, memory, attention, language, abstraction, evocation, and orientation. The maximum score is 30 points, with the cutoff point at 24 points.

#### Executive Functions

The Frontal Assessment Battery (FAB) [26] is a neurocognitive evaluation tool used to assess executive functions and has also been validated for the Portuguese population [27]. This test assesses six constructs: conceptualization, mental flexibility, programming, sensitivity to interference, inhibitory control, and environmental autonomy. The maximum score is 18 points, with three cutoff points: less than 12 indicates dementia, 12 to 14 indicates dysfunction, and 15 to 18 is in the normative range.

#### Memory

The Rey Complex Figure test (RCF) [28] was used to evaluate the capacity of motor perceptual organization, attention, and immediate nonverbal visual memory. This test is divided in two parts: the first part consists of an exercise in visual reproduction, followed by an interval of about three minutes, after which participants are asked to reproduce the same figure by memory (ie, the second part). The maximum total score of both parts is 36 points. Evaluation of the task is both quantitative (ie, performance time and points) and qualitative (ie, assessment of the level of reproduction). In this study we used only the quantitative scoring.

#### Attention

The Toulouse Pierón test (TP) [29] was used to evaluate permanent voluntary attention, concentration, resistance to fatigue, and stimulus processing. The test has a timed duration of exactly 10 minutes. During this time the participants have to identify the largest number of characters in a proof sheet from those that are indicated at the top of the sheet. The test measures the total number of characters processed (ie, hits) and omissions. The outcomes consist of correct responses, errors, and omissions along with dispersion index (DI) and working efficiency, which are given as follows:

Dispersion index = (errors + omissions) / hits × 100

Working efficiency = hits – (errors + omissions)

#### Cognitive Flexibility

The Wisconsin Card Sorting Test (WCST), developed by Grant and Berg [30] and studied by Nyhus and Barcelo [31], assesses strategic planning, abstract thinking, and capacity for perseveration and conceptualization. It also assesses the ability to use environmental feedback in modeling cognitive functioning, object-directed behaviors, and modeling of response impulsiveness. The test consists of 128 cards divided into two decks of 64 each. The cards vary in color (ie, red, green, yellow, and blue), shape (ie, triangles, stars, crosses, and circles), and number (ie, one, two, three, and four). Participants must match the 128 cards to the 4 stimulus cards that the examiner places on the table. The WCST was scored on six performance parameters: number of trials administered, number of total errors, number of perseverative errors, number of trials to complete first category, failure to maintain set, and conceptual-level responses.

### Procedure

#### Overview

The ethics committee of the School of Psychology and Life Sciences of the host institution approved the human subjects protocol used in this study. It was conducted according to best practices on human research and the principles of the Declaration of Helsinki.

After reading and signing the offline informed consent document, participants from both groups first completed a sociodemographic and clinical data questionnaire and then a flexible neuropsychological assessment battery consisting of tests of global cognition (ie, MoCA), executive functions (ie, FAB), attention (ie, TP), memory (ie, RCF), and cognitive flexibility (ie, WCST). Participants took between 1 and 1.5 hours to complete this session. Participants were given a code to ensure anonymity, and the questionnaires and battery of tests were identified with this code to pair them with posttreatment assessments.

Participants in the experimental group underwent the VR intervention plus treatment as usual during their second and third weeks of hospitalization. This intervention consisted of 10 sessions, each lasting 30 to 40 minutes, which ran twice a week over a period of 5 weeks; these sessions consisted of the performance of VR exercises based on activities of everyday life guided by one therapist in individual sessions, increasing in difficulty from session to session. Participants in the control group underwent treatment as usual for AUD.

The training intensity can be considered low in terms of the number of sessions, session duration, and total treatment dose, which fell below the common range of 10 to 14 hours [13]. These sessions were conducted at *Casa de Saúde do Telhal* during the patients’ psychological appointments for AUD recovery.

In the last week of hospitalization, both the control group and the experimental group participants completed the same battery of neuropsychological evaluation tests that were applied in the first session. This session was led by the same evaluator of the first assessment. None of the therapists or evaluators involved in these sessions were the owners of the software used.

#### Systemic Lisbon Battery

Cognitive training was conducted with the Systemic Lisbon Battery (SLB), release 2016 [19], a VR-based serious games platform that has been tested for cognitive training and assessment developed with Unity3D (Unity Technologies) for the Windows 10 system (Microsoft). Prior studies with the SLB for cognitive assessment have provided normative data with subsamples from the Portuguese population [19]. Regarding cognitive training, the SLB has been tested among different samples of participants with cognitive deficits due to brain injuries [32] and was also adapted to different technological platforms, such as mobile technology [33].

**Figure 1:**
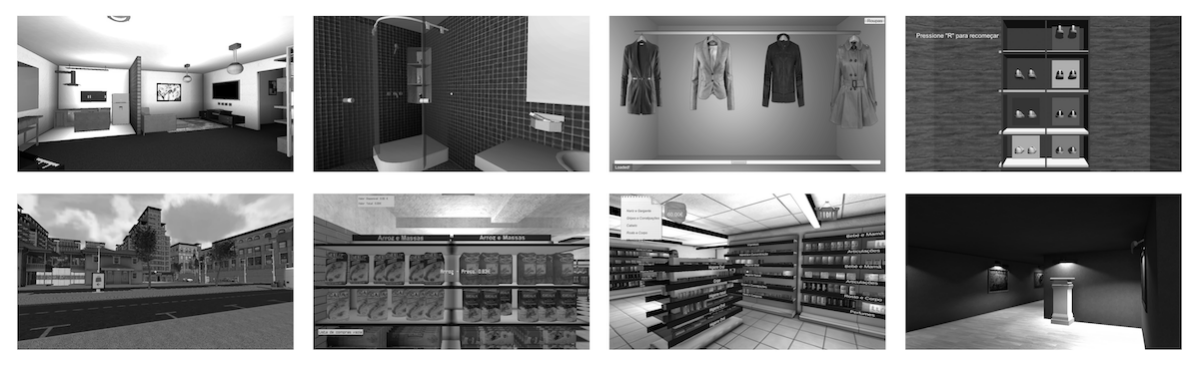
Examples of the virtual reality tasks used for cognitive training.

The SLB was developed as an alternative to the conventional methods of neurocognitive rehabilitation, but in this study it was used only for cognitive training. The platform consists of a virtual city with several built-up areas, a mini market, a pharmacy, an art gallery, and an interactive home; it also includes nonplayer characters walking around the city. The user is free to walk around the city and is given tasks to pick up certain objects in order to achieve a number of preset goals (eg, buy ingredients from a list at a grocery store). Elements from serious games, such as amount of money used or saved being used as a performance score along with visual and auditory feedback following completion of the tasks, are included in this platform to increase patients’ motivation and retention rates with therapy. Figure 1 depicts some of the tasks comprising the SLB.

### Statistical Analysis

The data were analyzed using SPSS Statistics for Windows, version 21.0 (IBM Corp). Normality was assessed by analyzing the distributions for each outcome according to skewness and kurtosis and was tested with the Shapiro-Wilk test. Skewness and kurtosis were within limits (±2) for the study variables, except for the DI of the TP. Shapiro-Wilk tests revealed that only the DI of the TP at both assessments and the copy trial from the RCF at postassessment violated the normality assumption. Therefore, those variables were assessed with nonparametric tests.

The baseline characteristics of the groups were compared using Student *t* tests for independent samples for outcomes with normal distribution and Mann-Whitney tests for independent samples for outcomes without normal distribution.

To test the effects of the treatment, we used repeated-measures analyses of covariance (ANCOVAs) with one within-subjects factor (ie, pretreatment vs posttreatment assessment) and one between-subjects factor (ie, experimental vs control group) while controlling for potential confounders. Effect sizes in the ANCOVAs are reported as η^2^ and are given by the following equation:

η^2^ = SSB / SST

where SSB is the sum of squares for between-subjects factors and SST is the sum of squares for the total model. Following Cohen [34], we consider a small effect size as starting from 0.01, medium as starting from 0.06, and large as starting from 0.14, so as to inform future definitive RCTs. The statistical tests were explored with post hoc tests using the Bonferroni correction method. The statistical results were tested with an analysis of simple effects using Bonferroni correction. For nonnormal distributions, Wilcoxon signed-rank tests for two related samples were conducted separately for the experimental group and the control group. The significance level was set at α=.05.

## Results

### User Statistics

**Figure 2:**
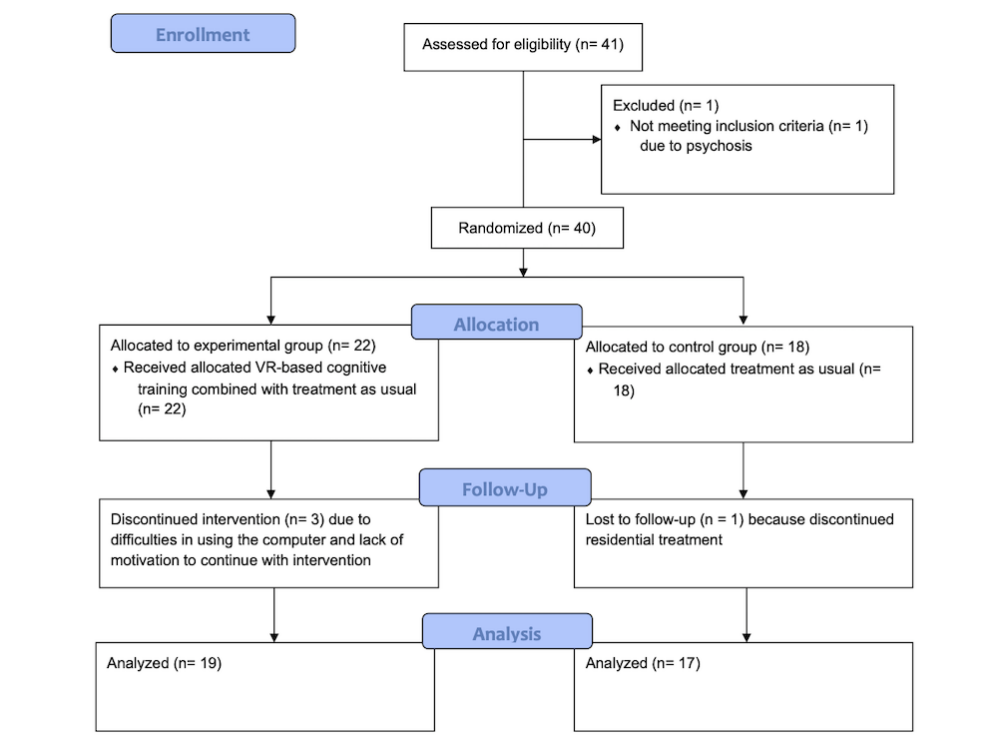
CONSORT (Consolidated Standards of Reporting Trials) 2010 flow diagram. VR: virtual reality.

The final sample consisted of 36 patients; 30 (83%) were male, they were aged between 24 and 65 years (mean 44.83, SD 12.04), and the mean number of years they consumed alcohol was 14.31 (SD 4.34). From those 36 patients, 19 (53%) were assigned to the experimental group and 17 (47%) were assigned to the control group. From the total sample of 36 participants, 5 (14%) reported having used other substances in addition to alcohol in the past. Out of 36 participants, 6 (17%) had completed only 4 years of school (ie, elementary school), 22 (61%) had completed 6 years of school (ie, middle school), 7 (17%) had completed 9 years of school (ie, high school), and 1 (3%) had completed more than 12 years of school (ie, a higher degree). No differences were found between the experimental and control groups regarding gender distribution (*P*=.29) or level of education (*P=*.38). However, age did differ between the experimental and control groups; it was significantly higher in the control group (mean 51 years, SD 12.34) than in the experimental group (mean 40 years, SD 9.21; *P*=.004). Years consuming alcohol did not correlate with neuropsychological performance in these tests. The initial pool consisted of 41 patients who were recruited for the study, but 1 (3%) was excluded based on the exclusion criteria, while 4 (11%) were lost to follow-up: 3 from the experimental group were lost due to difficulties in using the computer and a lack of motivation to continue with cognitive training and 1 from the control group had discontinued residential treatment (see flow diagram in Figure 2).

### Evaluation Outcomes

#### Baseline Comparisons Between Groups for Each Outcome

The comparisons between groups at the baseline assessment for each of the outcomes showed statistically significant differences in the total scores on the MoCA test (*t*_34_=3.049; *P*=.004) and the FAB test (*t*_34_=2.587; *P*=.01), suggesting better cognitive and executive functioning in the experimental group than in the control group.

However, because these differences could be due to the higher average age of controls, further analyses accounted for this by using the MoCA and FAB scores at baseline as covariates. Age did not correlate with MoCA (*P*=.62) or FAB scores (*P*=.58).

#### Pre-Post Comparisons for Each Outcome

Pre-post comparisons were performed using ANCOVAs with treatment assessment point (ie, time 1 vs time 2) as a within-subjects factor and treatment (ie, experimental vs control group) as a between-subjects factor controlling for baseline MoCA and FAB total scores on outcomes that were normally distributed. For outcomes that violated normal distribution, separate Wilcoxon tests were conducted for the experimental and control groups controlling these confounders. These analyses revealed a significant effect of the intervention on attention in two out of five of the TP outcomes and on cognitive flexibility in two out of six of the WCST outcomes. Improvements between pre- and posttreatment assessments in the experimental group were found for attention, concentration, cognitive flexibility, visual perception, and memory; these are discussed in more detail in the following three sections.

#### Attention and Concentration

The ANCOVA revealed a significant interaction effect between factors for correct responses (*F*_1,32_=19.512; η^2^=0.609; *P*<.001) and for working efficiency (*F*_1,32_=10.986; η^2^=0.343; *P*=.002). Simple effects (ie, Bonferroni corrected) showed a more pronounced improvement in both outcomes for the experimental group (*P*<.001). The same analysis comparing groups at each assessment point showed a difference between groups only at the posttreatment assessment for correct responses (*P*=.02) and for working efficiency (*P*=.03), as depicted in Table 1.

**Table 1:** Attention outcomes through the Toulouse Pierón test (TP).

Attention outcome	Experimental group score, mean (SE)		Control group score, mean (SE)		
	Pretreatment	Posttreatment	Pretreatment	Posttreatment	
TP no correct responses	128.47 (7.95)	192.76 (10.12)	140.17 (8.48)	155.85 (10.79)	
TP working efficiency	106.54 (8.40)	171.34 (17.57)	112.79 (8.96)	108.61 (18.73)	

Wilcoxon tests were conducted separately for the experimental and control groups, controlling for MoCA and FAB scores that were divided into two groups by median split. Therefore, eight Wilcoxon tests were conducted comparing pre-post assessments for the DI: experimental group MoCA score of 23 and below (ie, median) versus experimental group MoCA score above 23, and experimental group FAB score of 14 and below (ie, median) versus experimental group FAB score above 14; the same design was used for the control group. These comparisons showed significant differences between pre-post assessments only for the group below the median score for the FAB, revealing a decrease in the DI for both the experimental group (*Z*=–2.240; *P*=.03) and the control group (*Z*=–2.934; *P*=.003), thus suggesting no group effects in pre-post assessments of the DI.

#### Cognitive Flexibility

The ANCOVA for each of the WCST outcomes indicated a significant interaction effect for the total number of errors (*F*_1,28_=12.482; η^2^=0.446; *P*=.001) and the number of trials to complete the first category (*F*_1,28_=4.653; η^2^=0.166; *P*=.04). Simple effects (ie, Bonferroni corrected) suggested more pronounced improvements in these outcomes in the experimental group (*P*<.001). A difference between groups was observed at the pretreatment assessment for the number of trials to complete the first category (*P*=.02), while at the posttreatment assessment a difference was found for the total number of errors (*P*<.001). A main effect was also observed for the number of trials administered (*F*_1,28_=9.041; η^2^=0.244; *P*=.006), indicating an improvement from pre- to posttreatment assessment in both groups. These results are shown in Table 2.

**Table 2:** Cognitive flexibility outcomes through the Wisconsin Card Sorting Test (WCST).

Cognitive flexibility outcome	Experimental group score, mean (SE)		Control group score, mean (SE)	
	Pretreatment	Posttreatment	Pretreatment	Posttreatment
WCST no errors	74.72 (3.14)	52.43 (2.70)	71.79 (3.62)	72.45 (3.12)
WCST no trials to complete first category	40.31 (3.92)	15.86 (1.62)	23.95 (4.53)	12.89 (1.87)

#### Visual Perception and Memory

Despite the fact that the ANCOVA did not reveal a significant effect in the total score from the RCF for the memory trial, the analysis of simple effects (ie, Bonferroni corrected) showed a significant improvement between pre- and posttreatment assessments in memory ability for the experimental group (*P*=.002) (see Table 3). The copy trial from the RCF was assessed using Wilcoxon tests conducted separately for the experimental and control groups, controlling for the MoCA and FAB scores: experimental group MoCA score of 23 and below (ie, median) versus experimental group MoCA score above 23, and experimental group FAB score of 14 and below (ie, median) versus experimental group FAB score above 14; the same design was used for the control group. Only one comparison was significant in the control group with FAB scores below the median (*Z*=–2.684; *P*=.007), which indicates that there were no consistent intervention effects on this outcome.

**Table 3:** Memory outcomes through the Rey Complex Figure test (RCF).

Memory outcome	Experimental group score, mean (SE)			Control group score, mean (SE)	
	Pretreatment	Posttreatment	Pretreatment		Posttreatment
RCF memory trial	21.54 (4.92)	37.80 (5.68)	22.16 (5.24)		24.99 (6.06)

## Discussion

### Principal Findings

There is evidence that cognition improves during recovery treatment for AUD, while such improvements may be enhanced by the specific effects of cognitive training [10]. Moreover, the use of VR tasks for CCT is better suited to promote the transfer of skills to everyday living, as these tasks are closer to real-life activities than most of the exercises used in classic cognitive training [23]. The aim of this study was to determine the specific contributions to cognition of a multidomain CCT using VR among residential patients recovering from AUD. For that, a pilot RCT was carried out to study the feasibility of this approach in distinguishing the effects of treatment with CCT. RCTs are the emerging gold standard for unbiased testing of treatments and have been lacking from this area of research.

The results found in this study converge with previous research [19,20] in which patients with AUD who underwent cognitive training showed greater cognitive improvements, particularly in areas related to executive functions, than those who did not undergo any particular cognitive treatment. In this study, specific attention effects and cognitive flexibility effects (ie, executive functions) were found.

At the initial assessment, the two groups had different results in the MoCA and FAB measures, suggesting different levels of general cognitive ability and executive functions. In order to control for the effect of this difference on the outcomes, these variables were controlled in the pre-post analyses. In those analyses, we found significant differences in cognitive performance between pre- and posttreatment for both groups in attention, as assessed with the TP, and in cognitive flexibility, a component of executive functions, as assessed with the WCST; this shows evidence of the positive effects of the residential treatment plan and alcohol abstinence, confirming a robust finding in the literature [10,11]. In addition, it was found that effect sizes in these outcomes were large and were more pronounced in attention outcomes than in cognitive flexibility.

However, in-depth analyses show that there were significant differences between the groups at the final assessment point for the correct responses and DI of the TP and total number of errors in the WCST; however, in other indicators (ie, number of trials to complete the first category in the WCST) that showed improvements between pre- and postassessments, the experimental and control groups did not significantly differ. This pattern of results suggests that there were effects by the VR training sessions in the experimental group beyond simple abstinence-related effects; this also suggests that the task-specific learning in cognitive training promoted neuroplasticity more clearly than abstinence by itself, thus promoting more consistent improvements in attention and cognitive flexibility, as specific components of executive functioning. In fact, executive dysfunctions typically associated with the prefrontal cortex are among the most pronounced deficits due to alcohol abuse [5].

Patient retention was another positive feature of this intervention that speaks to its feasibility, as only 14% (3/22) of patients discontinued the cognitive training intervention, which is near the lower bound of the range of 8% to 41% found in a recent review of previous studies [13].

Overall, this study suggests that the use of VR scenarios is a feasible option to enhance cognitive recovery in patients with AUD, specifically at the level of attention, and that it may also support improvements in cognitive flexibility; these are important cognitive abilities also underlying decision making and retention in recovery programs for AUD [35].

### Limitations

One main limitation of this study was the small dose of the intervention. Given the time of stay for rehabilitation in residential treatment at the partner institution, it was not possible to extend rehabilitation over a longer intervention period. Thus, training intensity was inferior to most studies with cognitive rehabilitation [13], which may have compromised the ability of this trial to detect other positive intervention outcomes, enhancing a type II error. The small sample size and low power also enhanced a possible type II error, and replication with larger samples is still needed. On the other hand, the fact that this study was not double-blinded could have increased a type I error. The use of a passive control group consisting of treatment as usual, but without virtual training, precluded the use of blinding procedures to both patients and evaluators. It is important that further studies have an independent evaluator blinded to patient assignment.

### Conclusions

In this study, we found a positive impact of the VR training on the cognitive rehabilitation, particularly on attention and executive functions, of individuals with AUD. Although the residential treatment according to the Minnesota Model has as its main objective the promotion and maintenance of abstinence behavior in relation to alcohol, it can also promote a recovery from alcohol dependence. Such cognitive improvements may not only contribute to a better quality of life among patients but also to their social and family functioning and, therefore, their ability to maintain abstinence.

Future studies should focus on more general outcomes related to functionality, well-being, or quality of life to help understand whether such cognitive-focused approaches also contribute to overall psychological adjustment or whether there are far-transfer effects of skills to dissimilar tasks than those trained in the program. It is also worth studying whether these effects remain stable with time when assessed at longer follow-ups.

## Abbreviations

ANCOVA: analysis of covariance
AUD: alcohol use disorder
CCT: computerized cognitive training
DI: dispersion index
FAB: Frontal Assessment Battery
MoCA: Montreal Cognitive Assessment
RCF: Rey Complex Figure test
RCT: randomized controlled trial
SLB: Systemic Lisbon Battery
SSB: sum of squares for between-subjects factors
SST: sum of squares for the total model
SUD: substance use disorder
TP: Toulouse Pierón test
VR: virtual reality
WCST: Wisconsin Card Sorting Test

## References

[1] Savic M, Room R, Mugavin J, Pennay A, Livingston M (2016). Defining “drinking culture”: A critical review of its meaning and connotation in social research on alcohol problems. Drugs.

[2] Management of Substance Abuse Unit, Department of Mental Health and Substance Abuse, World Health Organization (2014). Global Status Report on Alcohol and Health 2014.

[3] Oscar-Berman M, Valmas M, Sawyer K, Ruiz S, Luhar R, Gravitz ZR (2014). Profiles of impaired, spared, and recovered neuropsychologic processes in alcoholism. Handb Clin Neurol.

[4] Sullivan EV, Pfefferbaum A (2005). Neurocircuitry in alcoholism: A substrate of disruption and repair. Psychopharmacology (Berl).

[5] Moselhy HF, Georgiou G, Kahn A (2001). Frontal lobe changes in alcoholism: A review of the literature. Alcohol Alcohol.

[6] Fernández-Serrano MJ, Pérez-García M, Schmidt Río-Valle J, Verdejo-García A (2010). Neuropsychological consequences of alcohol and drug abuse on different components of executive functions. J Psychopharmacol.

[7] Zahr NM, Kaufman KL, Harper CG (2011). Clinical and pathological features of alcohol-related brain damage. Nat Rev Neurol.

[8] Noël X, Brevers D, Bechara A (2013). A neurocognitive approach to understanding the neurobiology of addiction. Curr Opin Neurobiol.

[9] Fals-Stewart W, Schafer J, Lucente S, Rustine T, Brown L (1994). Neurobehavioral consequences of prolonged alcohol and substance abuse: A review of findings and treatment implications. Clin Psychol Rev.

[10] Manning V, Wanigaratne S, Best D, Hill RG, Reed LJ, Ball D, Marshall J, Gossop M, Strang J (2008). Changes in neuropsychological functioning during alcohol detoxification. Eur Addict Res.

[11] Durazzo TC, Mon A, Gazdzinski S, Meyerhoff DJ (2017). Regional brain volume changes in alcohol-dependent individuals during early abstinence: Associations with relapse following treatment. Addict Biol.

[12] Fals-Stewart W, Lam WKK (2010). Computer-assisted cognitive rehabilitation for the treatment of patients with substance use disorders: A randomized clinical trial. Exp Clin Psychopharmacol.

[13] Nixon SJ, Lewis B (2019). Cognitive training as a component of treatment of alcohol use disorder: A review. Neuropsychology.

[14] Clare L, Woods RT (2004). Cognitive training and cognitive rehabilitation for people with early-stage Alzheimer's disease: A review. Neuropsychol Rehabil.

[15] Wilson BA, Gracey F, Evans JJ, Bateman A (2009). Neuropsychological Rehabilitation: Theory, Models, Therapy and Outcome.

[16] Yohman JR, Schaeffer KW, Parsons OA (1988). Cognitive training in alcoholic men. J Consult Clin Psychol.

[17] Goldstein G, Haas GL, Shemansky WJ, Barnett B, Salmon-Cox S (2005). Rehabilitation during alcohol detoxication in comorbid neuropsychiatric patients. J Rehabil Res Dev.

[18] Peterson MA, Patterson B, Pillman BM, Battista MA (2002). Cognitive recovery following alcohol detoxification: A computerised remediation study. Neuropsychol Rehabil.

[19] Gamito P, Oliveira J, Brito R, Lopes P, Rodelo L, Pinto L, Morais D (2016). Evaluation of cognitive functions through the Systemic Lisbon Battery: Normative data. Methods Inf Med.

[20] Rupp CI, Kemmler G, Kurz M, Hinterhuber H, Fleischhacker WW (2012). Cognitive remediation therapy during treatment for alcohol dependence. J Stud Alcohol Drugs.

[21] Howard MC (2017). A meta-analysis and systematic literature review of virtual reality rehabilitation programs. Comput Human Behav.

[22] Parsons TD (2015). Virtual reality for enhanced ecological validity and experimental control in the clinical, affective and social neurosciences. Front Hum Neurosci.

[23] Doniger GM, Beeri MS, Bahar-Fuchs A, Gottlieb A, Tkachov A, Kenan H, Livny A, Bahat Y, Sharon H, Ben-Gal O, Cohen M, Zeilig G, Plotnik M (2018). Virtual reality-based cognitive-motor training for middle-aged adults at high Alzheimer's disease risk: A randomized controlled trial. Alzheimers Dement (N Y).

[24] Nasreddine ZS, Phillips NA, Bédirian V, Charbonneau S, Whitehead V, Collin I, Cummings JL, Chertkow H (2005). The Montreal Cognitive Assessment, MoCA: A brief screening tool for mild cognitive impairment. J Am Geriatr Soc.

[25] Freitas S, Simões MR, Alves L, Santana I (2011). Montreal Cognitive Assessment (MoCA): Normative study for the Portuguese population. J Clin Exp Neuropsychol.

[26] Dubois B, Slachevsky A, Litvan I, Pillon B (2000). The FAB: A Frontal Assessment Battery at bedside. Neurology.

[27] Lima CF, Meireles LP, Fonseca R, Castro SL, Garrett C (2008). The Frontal Assessment Battery (FAB) in Parkinson's disease and correlations with formal measures of executive functioning. J Neurol.

[28] Rey A (1941). L'examen psychologique dans les cas d'encéphalopathie traumatique. (Les problems). Arch Psychol (Geneve).

[29] Toulouse E, Piéron H (1986). Toulouse-Piéron: Prueba Perceptiva y de Atención. Manual.

[30] Grant DA, Berg E (1948). A behavioral analysis of degree of reinforcement and ease of shifting to new responses in a Weigl-type card-sorting problem. J Exp Psychol.

[31] Nyhus E, Barceló F (2009). The Wisconsin Card Sorting Test and the cognitive assessment of prefrontal executive functions: A critical update. Brain Cogn.

[32] Oliveira J, Gamito P, Lopes B, Silva AR, Galhordas J, Pereira E, Ramos E, Silva AP, Jorge Á, Fantasia A (2020). Computerized cognitive training using virtual reality on everyday life activities for patients recovering from stroke. Disabil Rehabil Assist Technol.

[33] Gamito P, Oliveira J, Lopes P, Brito R, Morais D, Caçoete C, Leandro A, Almeida T, Oliveira H (2017). Cognitive training through mHealth for individuals with substance use disorder. Methods Inf Med.

[34] Cohen J (1992). A power primer. Psychol Bull.

[35] Shulman M, Campbell A, Pavlicova M, Hu M, Aharonovich E, Nunes EV (2018). Cognitive functioning and treatment outcomes in a randomized controlled trial of internet-delivered drug and alcohol treatment. Am J Addict.

